# Overexpression of a mutant form of TGFBI/BIGH3 induces retinal degeneration in transgenic mice

**Published:** 2008-06-13

**Authors:** Mauro Bustamante, Andrea Tasinato, Fabienne Maurer, Ilhem Elkochairi, Mario G. Lepore, Yvan Arsenijevic, Thierry Pedrazzini, Francis L. Munier, Daniel F. Schorderet

**Affiliations:** 1 Institut de Recherche en Ophtalmologie, Sion, Switzerland,; 2 Service de Génétique Médicale, Lausanne, Switzerland; 3 Centre Hospitalier Universitaire Vaudois, Lausanne, Switzerland,; 4 Université de Lausanne, Lausanne, Switzerland,; 5 Département de Médecine, Lausanne, Switzerland; 6 Hôpital Jules-Gonin, Lausanne Switzerland,; 7 Faculté des Sciences du Vivant, Ecole Polytechnique Fédérale de Lausanne, Lausanne, Switzerland

## Abstract

**Purpose:**

Despite ubiquitous expression of the keratoepithelin (KE) protein encoded by the transforming growth factor beta induced/beta induced gene human clone 3 (*TGFBI/BIGH3*) gene, corneal dystrophies are restricted to the cornea, and no other tissues are affected. We investigated the role of *TGFBI/BIGH3* in Groenouw corneal dystrophies by generating transgenic mice overexpressing TGFBI/BIGH3 containing the R555W mutation.

**Methods:**

Transgenic animals expressing the Groenouw mutation of human *TGFBI/BIGH3* were generated using lentiviral vectors. The line expressed TGFBI/BIGH3 containing the R555W mutation under the control of the phosphoglycerate kinase (*PGK*) promoter. Expression of the transgene was monitored by Southern and western blotting and by RT–PCR. Electroretinogram analysis was performed and four mice were subjected to complete necroscopy.

**Results:**

Transgene expression was observed in different organs although without specific expression in the cornea. The overall morphology of the transgenic animals was not severely affected by KE overexpression. However, we observed an age-dependent retinal degeneration both functionally and histologically. Female-specific follicular hyperplasia in the spleen and increased levels of lipofuscin in the adrenal gland were also seen in transgenic animals.

**Conclusions:**

Cellular degeneration in the retina of transgenic animals suggest that perturbation of the transforming growth factor beta (TGFβ) family regulation may affect photoreceptor survival and may induce possible accelerated aging in several tissues. No corneal phenotype could be observed, probably due to the lack of transgene expression in this tissue.

## Introduction

Human corneal dystrophies (HCD) are rare hereditary diseases characterized by bilateral loss of corneal transparency due to the slow accumulation of amyloid as well as non-amyloid deposits. Symptoms may become present as early as birth, but more frequently they develop during adolescence and gradually progress throughout life. We and others have mapped four different forms of HCD to chromosome 5q31 [1] and subsequently recognized that mutations in the transforming growth factor beta-induced gene (*TGFBI/BIGH3*) were the causes of these diseases [2]. Although mutations at two specific amino acids (R124 and R555) are responsible for the great majority of the cases, isolated families have been described with mutations in other parts of the gene [3,4].

Studies of *TGFBI/BIGH3*-related HCD are of great interest, and while keratoepithelin (KE), the product of *TGFBI/BIGH3*, is ubiquitously expressed except in the brain, it is still not understood why KE deposits only occur in corneas [5]. In this report, we describe a transgenic mouse line expressing an R555W mutated form of human *TGFBI/BIGH3* in an attempt to generate a model for the Groenouw type I HCD. Extensive postmortem analyses included macroscopic and histopathologic analyses of organs and measures of the principal metabolic enzymes. Our data indicate that transgene expression of *TGFBI/BIGH3* induced an age-dependent retinal degeneration, associated with concomitant significantly attenuated cone and rod responses. Female animals also displayed a mild follicular hyperplasia in the spleen and lipofuscin expression in the adrenal glands, suggesting an accelerated aging process. No other histopathologic abnormalities were noticed in the transgenic animals compared to control mice. In particular, no transgenic RNA expression was observed in the corneas of these animals.

## Material and methods

### Lentivirus generation

Recombinant lentiviral particles containing a vesicular stomatitis virus G protein (VSV-G) envelop were produced by transient transfection of 293T cells as previously described [6]. Briefly, 293T cells were transfected with the different plasmids using calcium phosphate, the medium harvested 36 h later and centrifuged at 70,000x g. Viral supernatants were concentrated by a second ultracentrifugation at 70,000x g for 90 min at 4 °C. The viral pellets were resuspended in a minimal of volume of PBS containing 10 mg/ml bovine serum albumin (BSA). Aliquots of 5 µl were then stored at −70 °C until further use. Total particle concentration of the viral stocks was estimated by quantification of the p24 capsid protein using Retro-TEK HIV-1 p24 Antigen ELISA kit (ZeptoMetrixCorporation, Buffalo, New York).

High transduction efficiency was observed in corneal endothelial cells in vivo, using an HIV-1-derived lentivirus with a cytomegalovirus immediate early promoter (CMV). A similar efficacy was reported for rat, ovine, and human cornea explants infected in vitro with a lentiviral vector carrying a simian virus (SV) promoter [7,8]. Previous results have shown similar efficacy in mouse cornea using HIV or equine infectious anemia virus (EIAV) vectors containing the vectors containing the VSV-G envelop (VSVG) envelop and the CMV vector [9]. In cell culture, EIAV has also shown a strong efficiency to transduce human endothelial cells using a CMV promoter and the VSV-G envelop [10].

**Figure 1:**
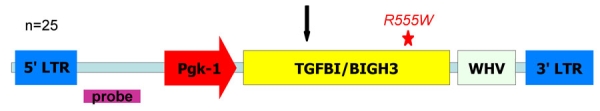
Lentiviral backbone containing the phosphoglycerate kinase promoter and the post-transcriptional regulatory element of woodchuck hepatitis virus. Note the Groenouw (R555W) mutation, the localization of the probe used for Southern blotting, and the unique BamHI site (black arrow). In the figure, n indicates the number of founders obtained for the construct.

A human *TGFBI/BIGH3* cDNA with the R555W Groenouw mutation was ligated into BamHI sites of a replication-deficient, self-inactivating plasmid (pSIN) under the regulation of the mouse phosphoglycerate kinase promoter (*PGK*; Figure 1). The *PGK* promoter is a ubiquitous promoter that was successfully used with lentiviral vectors in many tissues [11,12]. Since the GC-rich sequence of the mouse *PGK* promoter contains only three ATG triplets, this promoter is particularly suitable for lentiviral constructions as compared to other ubiquitous promoters [13]. Presence or absence of mutation was confirmed by direct sequencing of the insert.

### Lentiviral vector preparation

A replication-deficient, self-inactivating (SIN) backbone was obtained from Patrick Aebischer (Ecole Polytechnique Fédérale de Lausanne [EPFL], Lausanne, Switzerland). The transgene encoding the human *TGFBI/BIGH3* cDNA was inserted into a backbone containing the SIN followed by the elongation factor-1 minimum promoter and the woodchuck hepatitis post-transcriptional regulatory elements (SIN-W-EFs) to generate the lentiviral vector LV_BIGH3. The lentiviral vector was produced as described above and previously by Naldini [14].

### Generation of transgenic animals

All procedures implicating mice manipulation were performed in agreement with the ARVO Statement for the Use of Animals in Ophthalmic and Vision Research and were approved by the local Veterinary Committee Office on Use and Care of Animals in Research of Canton Vaud, Lausanne, Switzerland. Mice (120 total, 60 males and 60 females) were maintained on a 14:10 light-dark cycle under standard housing conditions. Water and standard high protein food (Kliba, Switzerland) were available at libitum.

Transgenesis was performed according to standard protocols [15]. Briefly, four-week-old National Marine Research Institute (NMRI) females were superovulated by intraperitoneal injection of 5U pregnant mare’s serum gonadotropin followed by injection of 5U human chorionic gonadotropin 48 h later. Superovulated females were mated with NMRI males, and embryos were collected at zygote stage on the following day. Zygotes were then immediately microinjected into the perivitelline space with 10-100 picoliters of about 109 transduction unit/ml lentiviral preparation and incubated in M16 medium (Sigma, St. Louis, MO) at 37 °C and 5% CO_2_ atmosphere. On the following day, two-cell stage embryos were selected and transferred into the oviduct of 3 pseudopregnant NMRI females.

NMRI mice are widely used as experimental models in many fields of general biology. Albino NMRI mice are widely used in transgenesis since embryos derived from this strain are more resistant than inbred mouse embryos such as C57/BL6 and their use results in a higher transgenesis rate. Moreover, outbred mice embryos are more resistant than inbred mice embryos and lead to a higher transgenesis rate.

### RNA extraction

Several organs including retina and cornea were removed from euthanized mice and directly placed in ice. Euthanasia was performed by neck dislocation. Total RNA was extracted using Trizol® reagent according to the manufacturer’s instructions (Invitrogen, Carlsbad, CA). RNA concentration and purity were measured using RiboGreen® (Molecular Probes, Eugene, OR) according to the manufacturer’s instructions.

### cDNA synthesis

Equal amounts (1 µg) of RNA were used to synthesize cDNA using StrataScript Reverse Transcriptase according to the manufacturer’s instructions (Stratagene, La Jolla, CA).

### Genomic DNA extraction

Genomic DNA was obtained from mouse tail biopsies as follows: cut tail was transferred into lysis buffer, which contained 100 mM TrisHCl, pH 8.5, 5 mM EDTA, 0.2% sodium dodecyl sulfate (SDS), 200 mM NaCl, and 100 µg proteinase K/ml, and incubated in a rotating shaker at 56 °C overnight. After complete lysis, samples were centrifuged for 2 min and the supernatant was transferred into isopropanol. After a second 2 min centrifugation, supernatant was removed and the pellet was dissolved in 150 µl 10 mM TrisHCl and 0.1 mM EDTA, pH 7.5, and incubated at 80 °C for 4 min. The obtained genomic DNA was directly used to perform PCRs.

### Transgene integration, expression, and monitoring

Transgene integration was determined from biopsies by PCR amplification of *TGFBI/BIGH3*. Genomic DNA (500 ng or 1 µg), obtained as described in the previous section, was amplified. If not otherwise stated, the conditions were as follows: a first incubation of 5 min at 94 °C, then 35 cycles of 1 min at 94 °C, 1 min at 56 °C and 3 min at 72 °C, and finally a last extension of 10 min at 72 °C. The following primers were used: Big-Big_F, Big-Big_R, PGK-BIG_F, and PGK-BIG_R (Table 1). Since the human transgene sequence contained a unique BamH1 site in a specific PCR-amplified region, transgene transcription was monitored by digestion of the PCR amplified cDNA produced by reverse-transcribed RNA. PCR was performed from 100 to 500 ng cDNA amplified by Hotstart Taq polymerase (Qiagen, Hilden, Germany), with the primers 227F and 649R (Table 1) and the following conditions: a first incubation of 5 min at 95 °C, then 30 cycles of 1 min at 95 °C, 1 min at 55 °C and 1 min at 72 °C, with a final extension of 10 min at 72 °C. BamHI digestion was performed from 0.1 to 1 µg gel purified PCR product incubated 8-16 h at 37 °C with 1 unit of enzyme in Roche B buffer. Digestion of endogenous mouse TGFBI/BIGH3 resulted in a product of about 500 bp whereas BamHI digestion of transgenic (human) TGFBI/BIGH3 resulted in two bands of about 300 bp and 200 bp.

**Table 1:** List of all the primers used in the generation of a transgenic mouse line.

**Primer name**	**5'->3'**
Big-Big_F	AGTCATCAGCTACGAGTGCT
Big-Big_R	CCCATTAGGATAGTGGTGG
PGK-BIG_F	GATCCATCCAGGCGGCCGTG
PGK-BIG_R	GAGGGTCATGCCGTGTTTCA
227F	ACTTCACCAACTGCAAGCAG
649R	TCTGGATGTTGGAATTCTGG
LTR1-biotin	CTTAATACTGACGCTCTCGCACCA
LC1	GACCCGGGAGATCTGATTTC
LC2	GATCTGATTTCAGTGGCACAG
LC3	AGTGGCACAGCAGTTACG
LTR2	CACTGCTAGAGATTTTCCAC
LTR3	CCACACTGACTAAAAGGGTC
LTR4	GTCTGAGGGATCTCTAGTT

This table indicates the primers used in the different steps of generating the transgenic mouse line. All the primers are indicated in a 5'-3' direction and in triplet sets.

The number of transgene integration sites was monitored by Southern blot. Genomic DNA obtained was digested with EcoRI overnight at 37 °C. The digested DNA was precipitated with 3M sodium acetate (pH 5.2)/EtOH on ice and resuspended in trishydroxymethylaminomethane – ethylene diamine tetraacetic acid (TE) buffer. A full-length *TGFBI/BIGH3* cDNA was radiolabeled with the random primed technique and hybridized to the membrane. Hybridization was performed with a probe from the vector’s backbone (Figure 1). Integration sites were also monitored by linear amplification mediated polymerase chain reaction (LAM-PCR) as described [16]. Briefly, a linear PCR with a long-terminal repeat (LTR)-specific biotinylated primer (LTR1 in Table 1) was performed. The specific amplified PCR products were sorted by magnetic tag selection of extension primers. A second DNA strand of each target sequence was synthesized by random hexanucleotide priming. Double-stranded DNA was digested with CfoI, and an asymmetric ligation cassette was ligated to the end of CfoI-digested fragments. Nested exponential PCR amplifications were then performed with linker cassette (LC)-specific forward primers (LC primers, Table 1) and LTR-specific reverse primers (LTR primers Table 1). Primer pairs were LC1-LTR2, LC2-LTR3, and LC3-LTR4. Finally, LAM-PCR products were subcloned in pGEM-T easy vector, sequenced, and blasted against mouse genomic DNA.

### Protein extraction

Retina and cornea were removed from dissected mouse eyes. Proteins were extracted and homogenized for 10 min in 100 µl ice-cold RIPA buffer, which consisted of 50 mM Tris-Cl pH 8.0, 150 mM NaCl, 1% NP-40, 0.5% deoxycholate, and 0.1% SDS, and also contained protease (Roche, Mannheim, Germany) and 1% phosphatase (Sigma) inhibitor cocktails. Further homogenization through a needle was performed when necessary. Retinas or corneas were homogenized in ice-cold RIPA buffer with an 18 gauge needle. Protein concentrations were measured by bisinchoninic acid (BCA) protein assay (Pierce, Rockford, IL).

### Western blot analysis

Equal amounts of protein samples were separated by 8% SDS–PAGE gel and transferred to a polyvinylidene difluoride membrane (Westran clear signal; Whatman Inc., Maidstone, UK). Membranes were then blocked for 1 h at room temperature with Tris-buffered saline, composed of 150 mM NaCl and 50 mM Tris, pH 7.5, and contained 0.1% Tween (TBS-T) and 5% nonfat dry milk. Next, membranes were probed with a polyclonal rabbit antiserum against human KE-2 (1/4,000) [17] for 2 h in TBS-T with 5% nonfat dry milk. A secondary horseradish peroxydase-coupled goat antirabbit IgG (1/25,000; Amersham Biosciences, Buckinghamshire, UK) was used for 1 h at room temperature in TBS-T with 5% nonfat dry milk. After three washes in TBS-T, the antigen-antibody complexes were detected by chemiluminescence using the Enhanced Chemi Luminescence Plus (ECL+) western blotting system (Amersham Biosciences). Results were quantified under conditions of linearity by integration of the density of total area of each band using a video densitometer and LabWorks 4.0 software (Media Cybernetics, Bethesda, MD). Results are expressed as a percentage of the control optical density.

### Hematoxylin-eosin and DAPI staining of mouse eyes

Mouse eyes (8 transgenic and 3 wild type) were dissected and fixed in 4% paraformaldehyde for 1 h. Fixed eyes were then embedded in 30% albumin egg, 3% gelatin (Yazzula buffer) and frozen in liquid nitrogen to be sectioned at 14 µm with a cryostat. Slides were rehydrated and stained with hematoxylin for 10 min. After two washes, slides were stained with eosin for 1 min and fixed in 50% glycerol-50% PBS.

Different slides were stained for 15 min with DAPI safe from the light and then fixed with FluorSave. In order to analyze similar fields, we evaluated slides on which the optical nerve was visible on a BX51 Olympus microscope at 100X or 400X magnification. Pictures were taken using Cell^D^ imaging software (Carl Zeiss, Jena, Germany).

### Autopsy

Eight-month-old transgenic mice (two males and two females) and wild-type (one male and one female) living mice were sent to Frimorfo Inc. (Fribourg, Switzerland) for an in depth analysis of all organs. This analysis includes macroscopic inspection, necroscopy and histopathological evaluation completed by full X-ray analysis and blood analysis (blood chemistry and hematology).

## Results

### Generation and validation of the transgenic model

**Figure 2:**
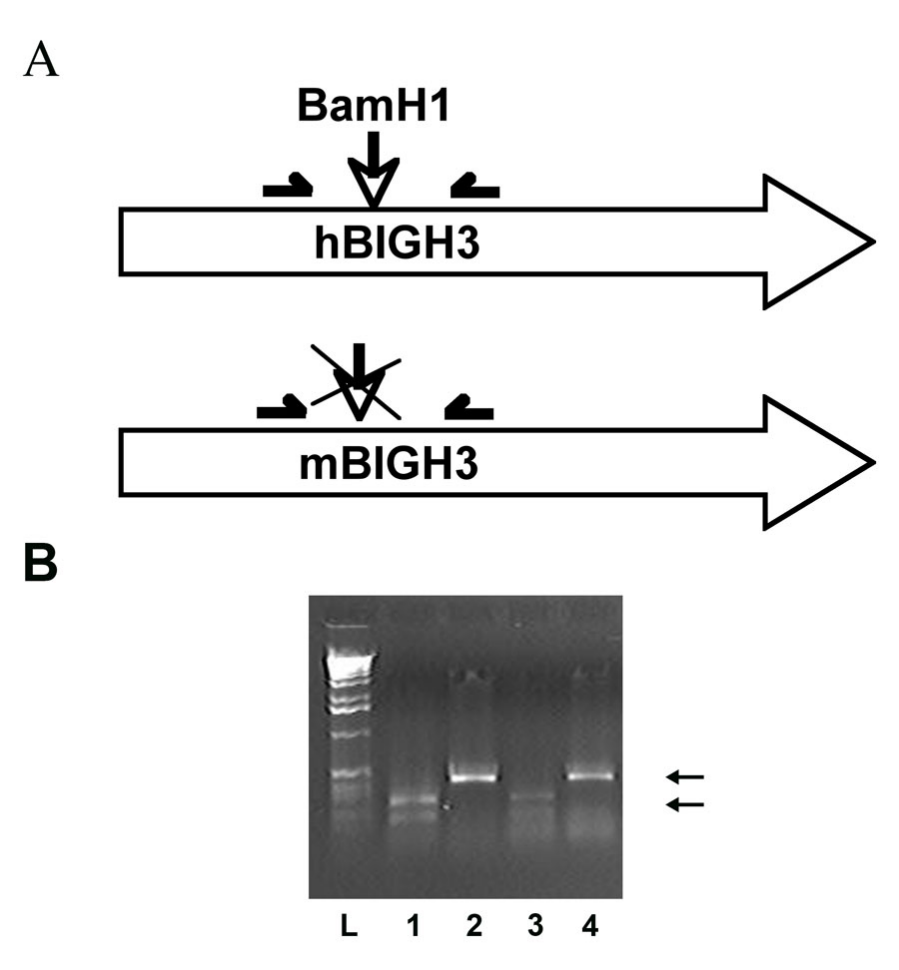
Screening for transgenic *TGFBI/BIGH3* mRNA expression. **A:** The scheme shows the difference between human and murine *TGFBI/BIGH3*. Note the localization of the primers used for the PCR and the unique BamHI site present only in the human gene. **B:** Migration of undigested corneal (lanes 2 and 4) or BamHI digested retinal (lanes 1 and 3) cDNA amplimers from two different PGK-Groenouw transgenic mice, as showed in **A**. L indicates the 1-kb DNA ladder lane.

In total, 25 independent PGK-Groenouw transgenic founders were generated, with a transgene integration averaged ratio of 37%±18 of the newborns, depending on the lentiviral batch and titer. In this study, we observed that only batches of lentiviral particles titrated above 1x10^8^ TU/ml were effective in generating transgenic animals. We routinely screened the mice by PCR for transgene integration, using primers specific for the transgene and RT–PCR for transcript expression (Figure 2). As described in the previous section, digestion of endogenous mouse *TGFBI/BIGH3* resulted in only one band whereas BamHI digestion of transgenic (human) *TGFBI/BIGH3* resulted in two bands.

**Figure 3:**
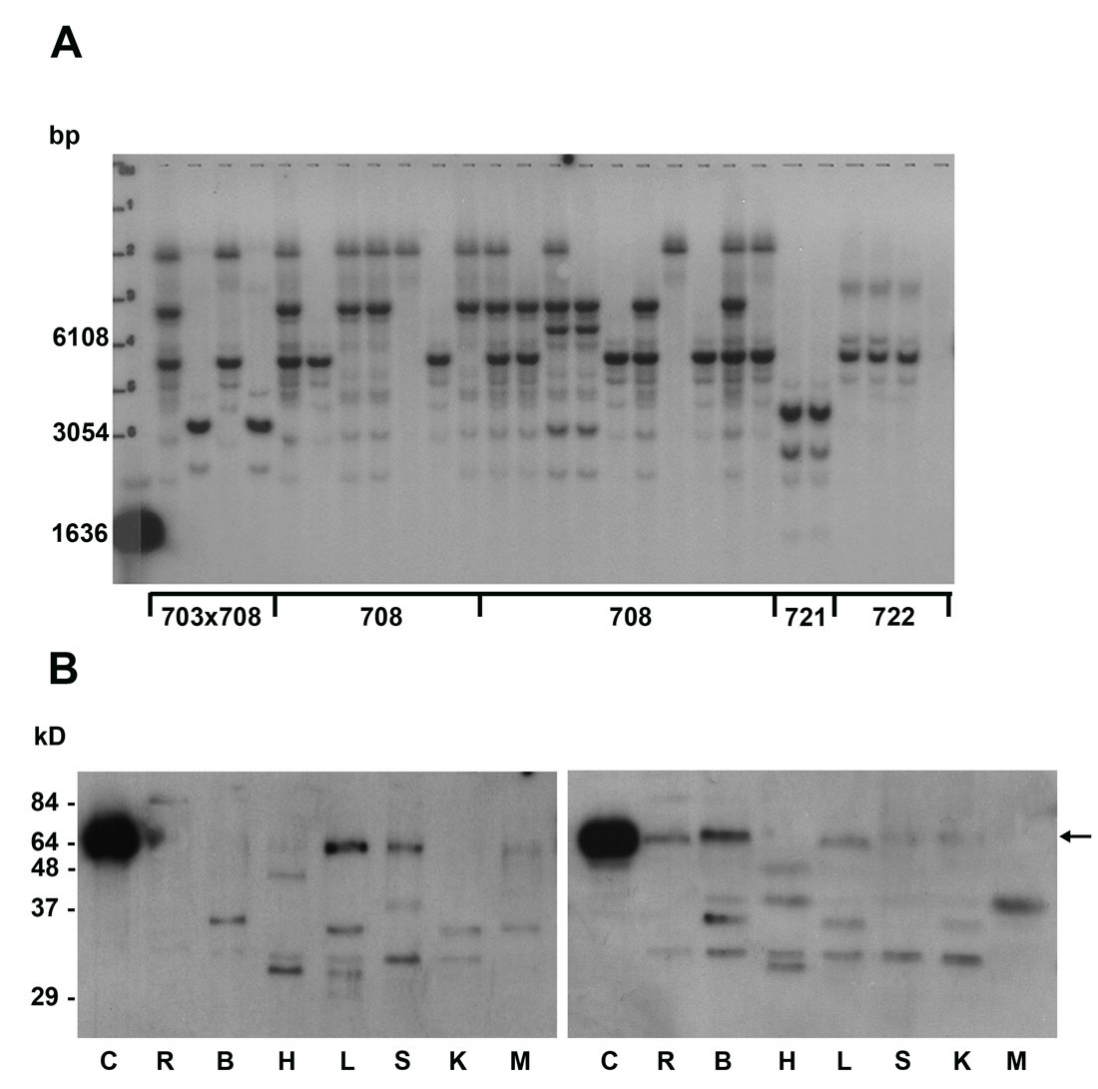
*TGFBI/BIGH3* expression analyzed by Southern and western blots. **A:** The Southern blot shows various integration sites of the transgene in 27 PGK-Groenouw F1 transgenic animals originating from four different founders. **B:** The Western blot against TGFBI/BIGH3 of the wild type (left panel) shows no expression in the retina and the brain in comparison with the western blot of a PGK-Groenouw transgenic mouse (right panel). The following abbreviations are used in (**B**): cornea (C ), retina (R ), brain (B), heart (H), liver (L), spleen (S), kidney (K), and muscle (M). The arrow indicates 64 kDa band corresponding to keratoepithelin.

Southern blot analyses were used to evaluate the number of integration sites in the F1 generation of transgenic mice. Generally one to four sites were observed (Figure 3A). Based on the size of the fragments, and assuming no recombination in the region of the probe, five integration sites were seen in the Southern blot analysis of transgenic mice. It is assumed that, with successive generations, the number of integration sites will diminish. Moreover, localization of integration sites were monitored in some transgenic animals using linear amplification mediated polymerase chain reaction (LAM-PCR) method. Integration of lentiviral vectors were seen in chromosomes 7, 8, 14, 19, and Y. Table 2 indicates the exact positions of the identified integration sites.

**Table 2:** Location of virus integration sites in transgenic mice

**Chromosome**	**Contig**	**Position**	**Mouse ID**
7	NT_039407.1	1329488	708, 718
			
8	NT_081820.1	303322	705, 724
			
8	NT_039456.3	12023612	703, 708
			718, M15
			M129, M130
			M139, M142
			M165, M172
			M176, M178
			M179, M191
			M196
			
14	NT_039606.7	34668	708
			
19	NT_039689.1	3483949	708

17 different mice (Mouse ID) have been submitted to linear amplification mediated polymerase chain reaction (LAM-PCR). Five different integration sites have been identified, and each of them is characterized by its chromosome, its contig and its exact position.

We additionally monitored tissue expression in glycerol-embedded sectioned tissues (not shown) and by western blot using an anti-KE2 antibody in transgenic mice expressing the R555W mutation under the control of the *PGK* promoter. KE expression was slightly increased in the retina of transgenic mice compared to wild-type mice (Figure 3B, left panel). We also observed a clear expression of KE in the brain of transgenic mice, whereas no expression was found in wild-type mice (Figure 3B). Overall, it did not seem to affect endogenous expression in other tissues (Figure 3B, left and right panels). The figure also showed smaller bands corresponding to degradation products of the protein.

### Organ comparisons between phosphoglycerate kinase promoter-Groenouw transgenic and wild-type mice

The weight of the organs of eight-month-old transgenic mice (two males and two females) was compared to age- and sex-matched control mice. Transgenic mice appeared to be healthy and did not display particular abnormal behavioral signs for up to 18 months.

**Figure 4:**
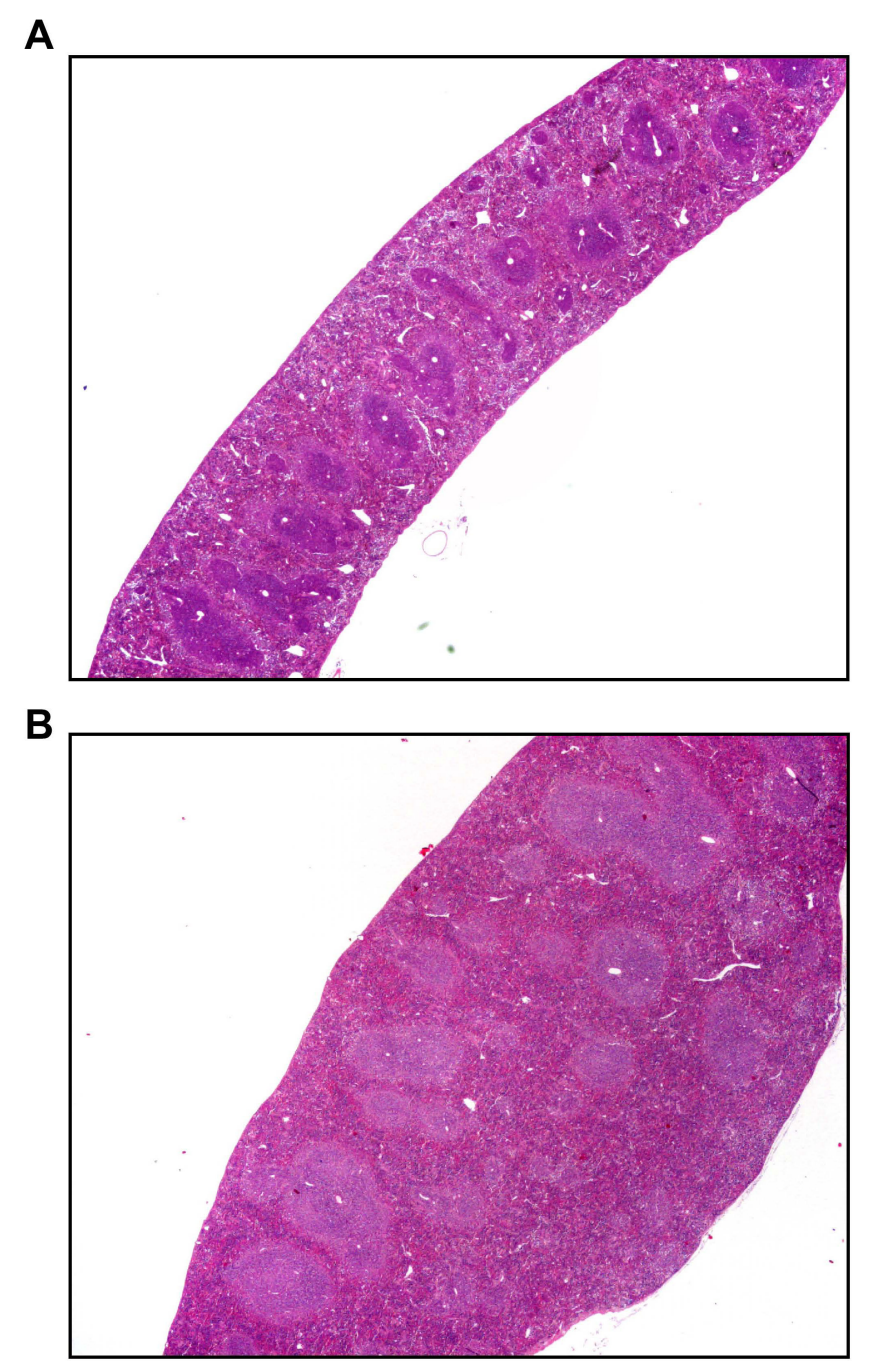
Hyperplasia histology analysis of the spleen in a transgenic mouse compared to a wild type mouse. Shown is a hematoxylin-eosin coloration of spleen taken from an eight-month-old wild type (**A**) mouse and an eight-month-old transgenic (**B**) mouse. Hyperplasia of the folliculi in the transgenic mice can be seen in (**B**). Both pictures were taken at 100X magnification.

Transgenic mice tended to have increased organ size and weight, except for the thymus that was reduced in size compared to age-matched wild-type NMRI mice (Table 3). Transgenic female mice showed follicular hyperplasia (Figure 4, lower panel), increased amount of extramedullary hematopoiesis, and higher spleen weights (Table 3). The weight of the heart and kidneys was higher in transgenic females (Table 3), but morphology was normal.

**Table 3:** Mean organ weight of transgenic and wild-type mice.

**Organ**	**Mean weight (grams)**	
	**Transgenic (SEM)**	**Control (SEM)**
Body weight	46.65 (14.84)	38.85 (10.82)
Brain	1.21 (0.46)	1.245 (0.40)
Spleen	0.39 (0.22)	0.26 (0.07)
Liver	5.405 (1.62)	4.07 (0.30)
Left Kidney	0.94* (0.13)	0.675 (0.02)
Right Kidney	0.935* (0.10)	0.675 (0.06)
Heart	0.505 (0.12)	0.39 (0.03)
Thymus	0.06 (0.04)	0.08 (0.04)

Four transgenic and two wild-type mice were sacrificed and weighed. SEM indicates the standard deviation observed for each organs, and asterisk indicate significant differences between transgenic and wild-type organ (the asterisk denotes a p<0.05).

We did not observe other differences between males and females for relative weight or histology of other organs, compared to non-transgenic animals. Overall, no statistical differences were observed in protein concentrations (Table 4A) or in cell composition (Table 4B) of blood between transgenic mice and wild-type mice. One transgenic female presented lipogenic pigment accumulation in the adrenal gland.

**Table 4:** Blood chemistry and hematology of transgenic and wild-type mice.

**A**		
**Blood Chemistry**	**Transgenic**	**Control**
Serum Glucose (mmol/l)	9.08	7.60
Urea (mmol/l)	11.67	9.15
Creatinine (μmol/l)	25.00	18.00
Protein (Biuret; g/l)	69.37	49.65
Albumin (g/l)	41.44	30.35
Cholesterin (mmol/l)	5.07	3.10
ASAT (GOT; U/l)	90.33	63.50
ALAT (GPT; U/l)	50.33	30.50
CK (U/l)	376.00	220.50
Amylase (U/l)	4707.27	3376.70
Calcium (mmol/l)	3.07	2.24
Phosphate (mmol/l)	2.46	1.67
Sodium (mmol/l)	209.60	156.85
Potassium (mmol/l)	6.09	4.45
**B**		
**Hematology**	**Transgenic**	**Control**
Hematocrit (%)	0.455	0.43
Hemoglobin (g/l)	161.5	158
Erythrocytes (x10^12^/l)	9.53	9.32
Leukocytes (x10^9^/l)	5	5.3
MCH (pg)	16.75	17
MCHC (g/l)	354.5	367
MCV (fl)	48	46
Thrombocytes (x10^9^/cl)	13.96	12.45
Banded Neutrophils (%)	0.375	0.25
Segmented Neutrophils (%)	22.5	12.75
Eosinophils (%)	2.125	1.25
Monocytes (%)	1.125	1
Lymphocytes (%)	73.875	84.75
Segmented Neutrophils (x10^9^/l)	1.2	0.71
Eosinophils (x10^9^/l)	0.1275	0.06
Lymphocytes (x10^9^/l)	3.5725	4.445

Blood was removed from four transgenic and two wild-type mice. Mean measures have been measured for blood chemistry (**A**) and hematology (**B**) in transgenic and wild-type mice. Abbreviations in the table: ASAT is aspartate amino transferase; ALAT is alanine amino transferase; CK is creatinine kinase; MCH is mean corpuscular hemoglobin; MCHC is mean corpuscular hemoglobin concentration; MCV is mean corpuscular volume.

### Transgenic-dependent retinal degeneration

**Figure 5:**
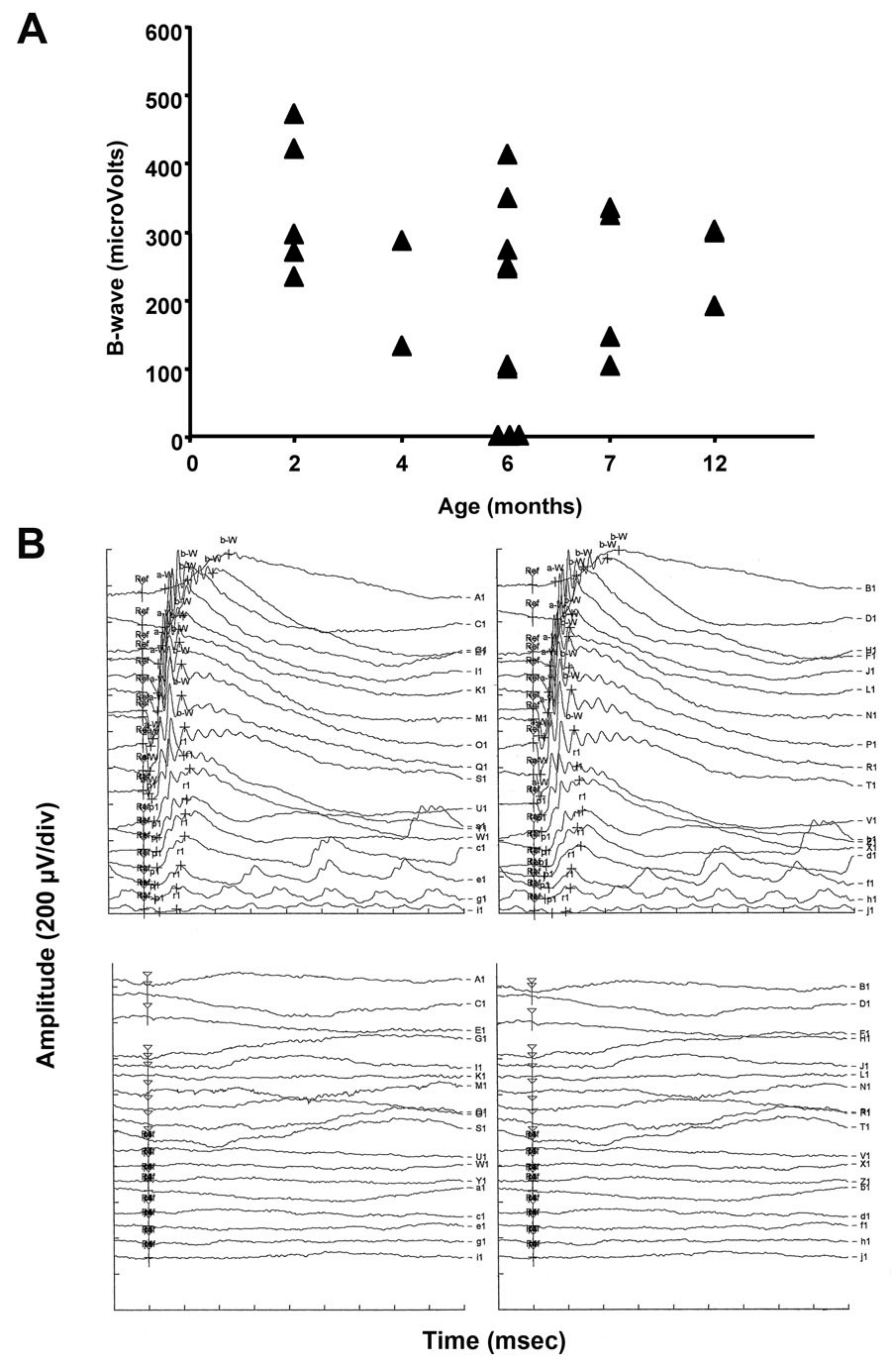
Electroretinogram analysis of transgenic mice at various ages. **A:** Amplitudes of b-waves of PGK-Groenouw transgenic mice’s electroretinogram (ERG) distributed in function of their age. **B:** ERG recordings in response to single flashes in scotopic conditions. Two different six-month-old PGK-BIG Groenouw transgenic animals showed either a normal response (upper panel) or a flat ERG (lower panel). Conditions: A1 to V1 shows records with increasing intensities of light from 0.1 to 25000 mcds/m^2^ and each division of the time axis correspond to 40 ms. U1 to j1 shows records with increasing frequencies of flashlight from 0.5 to 30 Hz and each division of the time axis correspond to 50 ms.

**Figure 6:**
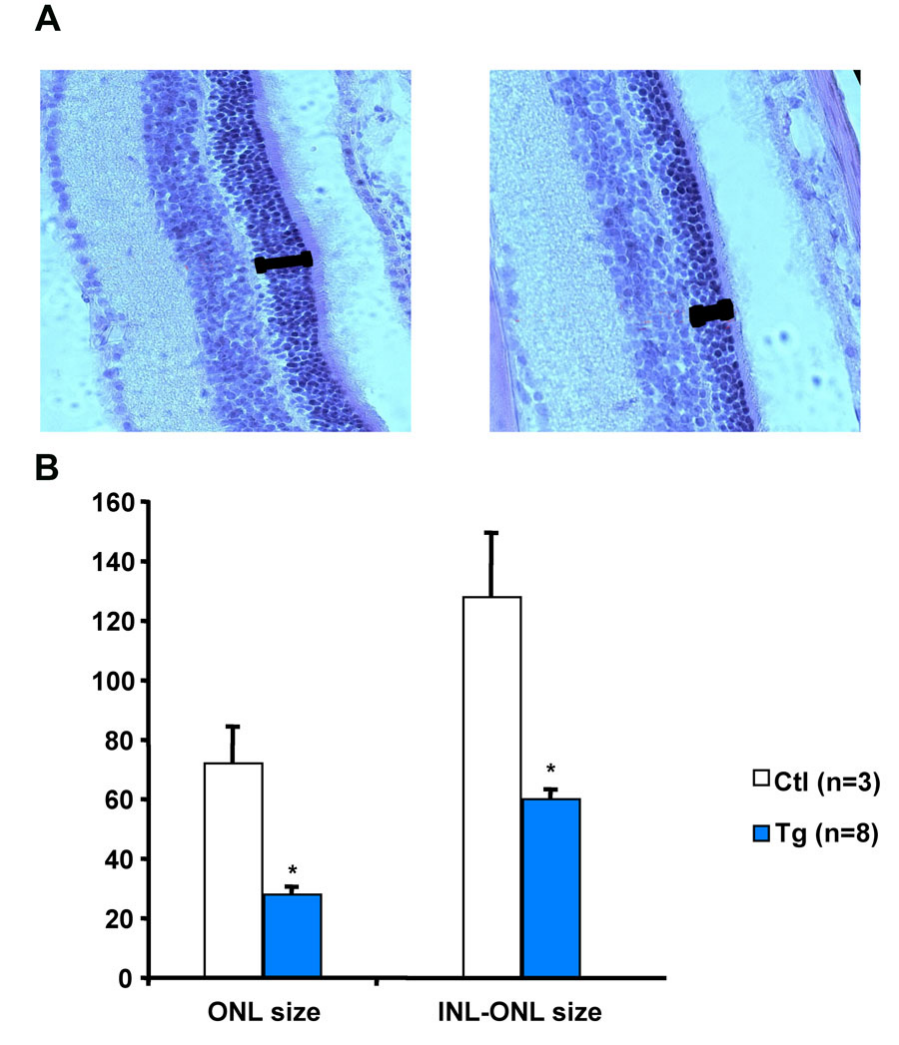
Measurement of the outer nuclear layer of the retina in transgenic and wild-type mice. **A:** Retinas of a wild type (left panel) and of a PGK-Groenouw transgenic (right panel) mouse stained with hematoxilin-eosin. The bars indicate the outer nuclear layer (ONL) of the retina. The pictures were taken at a 600X magnification. **B:** ONL and outer plus inner nuclear layer (INL-ONL) of retinas from control nontransgenic (Ctl) and PGK-Groenouw transgenic (Tg) mice at 16 months of age were measured and quantified in the graph.

Transgenic expression of KE was observed in the retina but not in the cornea of transgenic animals. We therefore investigated the effect of *TGFBI/BIGH3* overexpression in retinal tissue. While electroretinogram (ERG) response to increasing intensities flashlights were normal in 2-month-old transgenic mice, we observed a reduction in a- and b-waves in more than 50% of the older mice (Figure 5). In scotopic conditions, at an illumination of 100 mcds/s, 2-month-old mice showed a mean b-wave of 346 mV whereas this mean measure falls to 211 mV at 6 month. Four out six (66.7%) mice showed b-waves even lower than the mean measure. In comparison and in the same conditions, NMRI wild type mice showed no difference in mean values from 2 to 6.5 month of age. Mice with flat ERG also showed a diminution of the retina thickness and a global disorganization of the cell disposition in the retina of transgenic mice expressing the Groenouw mutation compared to control mice (Figure 6). Quantifications of the outer and inner nuclear layers thickness showed a significant diminution of each layer corresponding to a global diminution in transgenic mice (Figure 6B).

## Discussion

Despite many studies on *TGFBI/BIGH3* in the development of human corneal dystrophy, its physiologic role is not yet entirely understood. To further elucidate the function of the gene, we generated a transgenic mouse line expressing one of the major mutant forms of human *TGFBI/BIGH3*.

*TGFBI/BIGH3* expression in our transgenic mouse line showed variable transgene amount expressed in different organs. However, our results consistently showed not only a clear overexpression of the protein in the heart and the kidney but a protein expression appeared in the retina and in the brain (Figure 3). Transgene expression of human *TGFBI/BIGH3* was also observed in other organs, but to a lesser extent.

Usually, viral vectors are known to randomly integrate in the host genome. However, a report by Hacein-Bey-Abina and colleagues [18] challenged this assumption, and the idea of preferred integration sites for viral vectors emerged. The integration sites varied, depending on the type of virus implicated, and occurred within active transcription units and more particularly, within or near genes encoding for proteins with kinase, transferase function, or phosphorylation activity [19,20]. As seen in Figure 3, several transgenic mice shared a same integration site on chromosome 8 (Table 2). We didn’t find any gene with retina-specific transcript in this region. The technique used did not allow us to identify all integration sites, and it is likely that other chromosomes regions harbored transgenes. This is corroborated by the results of the Southern blots showing more than two sites of integration.

Transgenic animals did not present apparent macroscopic abnormalities compared to control mice, suggesting that KE overexpression did not severely impair mouse development. Previous studies showed that *TGFBI/BIGH3* is involved in cell growth, tumor genesis, wound healing, apoptosis, migration, and osteogenesis among others [21]. This could mean that such actions are under the control of domains not mutated in our experiments. Results of blood analysis suggested that no metabolic change occurred in transgenic mice (Table 4). Moreover, no evident differences were observed at the cellular level except for spleen follicular hyperplasia in the transgenic female animals (Figure 4). We did not obtain any measurable KE expression in the cornea of transgenic animals with the promoter tested (Figure 3). This lack of expression was not due to transcriptional silencing since no transgene insertion was observed in DNA extracted from corneas (Figure 2). A possible explanation could be the systematic elimination of corneal cells overexpressing the transgene. Alternatively, this could be the result of an embryonic selection of corneal cells devoid of transgene, since we previously described that overexpression of KE induce apoptosis in cell lines [22]. It is possible that the KE silencing in the cornea might be consecutive to the method used to generate transgenic animals. Such a selection process may not be present later in life as Bainbridge et al. [7] showed stable transgenic expression in corneal endothelium when the transgene was directly injected in the anterior chamber. A transgenic mouse generated with the traditional method might also generate another expression pattern.

Interestingly, we observed an age-dependent retinal degeneration in transgenic mice that was not seen in wild-type controls (Figure 6). The observation was confirmed by ERG analyses (Figure 5). Moreover, lipogenic pigments were also observed in transgenic mice. This pigment is common in normal aging mice, but rare before 18 months of age. The presence of this pigment in our transgenic mice suggests an accelerated aging process that may explain in part the retinal degeneration. The follicular hyperplasia observed in the spleen was found only in female mutants. Again, this finding is relatively common in 6-month-old wild-type mice and increases in frequency with age, which is consistent with an accelerated aging process. A study by Kuro-o et al. [23] described a transgenic mouse line leading to a syndrome resembling human aging. The results showed a short lifespan, infertility, and arteriosclerosis, among others. Interestingly, the authors also noted a slight increase in calcium and phosphorus in blood serum, accompanied with a diminution of leukocytes and lymphocytes. We also observed this tendency in our data (Table 4) but our results are not statistically significant (p=0.59 and 0.65 for difference in calcium and lymphocyte concentrations respectively). This study also noted growth retardation in transgenic mice, which is in accordance with our results. On the other hand, Olsson et al. [24] showed that overexpression of normal proteins can damage photoreceptors because of protein trafficking abnormalities. This observation may be in accordance with the retinal degeneration observed in transgenic mice, but does not explain the early presence of lipogenic pigment and the follicular hyperplasia.

In conclusion, we generated a transgenic mouse line expressing an R555W human mutated *TGFBI/BIGH3*. Extensive histopathological evaluation, completed by full X-ray and blood analysis showed no evident differences compared to wild-type mice, except for retinal degeneration. The transgenic mice seemed to grow quicker than wild-type mice, as they tended to be bigger than age-matched wild-type mice. The presence of lipogenic pigment, the retinal degeneration, and the follicular hyperplasia of the spleen observed in the transgenic mice suggest an accelerated aging process.
